# Anti-Tuberculosis Organisation in London

**Published:** 1915-06-26

**Authors:** 


### ANTI-TUBERCULOSIS ORGANISATION IN LONDON.

### A Comprehensive Scheme for Adults and Children.

Organisation is most decidedly called for in dealing with tuberculosis, especially in the County of London, in view of the existence of a multitude of agencies and authorities through whose hands cases of tuberculosis may pass. The features peculiar to the disease commonly called consumption are of such a nature as to complicate very considerably the problem of dealing with its victims. Being both infectious and very chronic in its course, the pulmonary form of tuberculosis is a disease which taxes to the utmost the administrative forces of State medicine.

One of the first to devote a great deal of attention to the intricate problem of devising a scheme for the adequate control of tuberculous persons in the County of London was Dr. Barty King. Chiefly through his efforts a tuberculosis dispensary was established in connection with the out-patient department of the Royal Hospital for Diseases of the Chest, in the City Road. This dispensary, as was recorded in our columns, became the official tuberculosis dispensary for the Borough of Shoreditch. Since then several other hospitals have inaugurated departments for dealing with the treatment and prevention of tuberculosis of the lungs.

At St. Thomas's Hospital a branch dispensary has been established for some time, and this serves the Borough of Lambeth. Similarly, departments have been set going at St. Bartholomew's Hospital, at Charing Cross Hospital, and, more recently still, at the Metropolitan Hospital and at the Victoria Park Chest Hospital. Other schemes are now under consideration, and these, together with the many voluntary tuberculosis dispensaries of the type originally started in Paddington, will shortly provide centres for dealing with tuberculosis in every one of the twenty-eight Metropolitan boroughs and in the City. The deficiencies which still exist in certain districts would probably have been eliminated by now had it not been for the financial questions resulting from the war. These gaps in the network of agencies working against tuberculosis are something of a hindrance to the consummation of a satisfactory scheme, for London patients are rather prone to migration, and an essential part of any scheme for the adequate treatment of cases of pulmonary tuberculosis must be a means of co-operation and co-ordination of agencies, so that the desirable treatment in any individual case may be continuously obtainable.

Whatever may be the ultimate verdict on the value of, for instance, treatment by injections of tuberculin or by the production of an artificial pneumothorax, it is obvious that if, through change of residence, a patient finds it too difficult or impossible to obtain a continuation of such treatment, the results of such methods are likely to be disappointing. This is particularly the case when a patient has courageously submitted to the operation of having the chest filled with nitrogen on several occasions while undergoing sanatorium treatment. The value of this treatment depends entirely on the careful and repeated fillings with gas at intervals over a very long period, and so it should be provided that means for achieving this purpose be made available in many districts, if not all, in London. Otherwise it can hardly be expected that medical officers in sanatoria will deem it worth while to commence a somewhat heroic treatment if they see that there is little or no prospect of the patient being able to keep it up.

It will therefore readily be recognised that there is an insistent demand for a workable scheme to deal with tuberculous patients in the County of London, and Dr. Barty King has put himself to great pains to formulate such a scheme. In a book* completed before the London County Council scheme was passed, but only recently published, Dr. King has gone into very great detail in describing a comprehensive scheme. At first sight the book, with its exceedingly complex diagrams, looks too formidable even partially to be studied. Sir William Osler himself admits in his foreword that it looks complicated, but the scheme itself is comparatively simple, and does but consist in the co-operation of existing agencies. Unfortunately, by means of an excessive zeal in tabulation and sub-division the matter is to a large extent robbed of interest for the average tuberculosis officer or hospital physician. The charts themselves are the most flagrant examples of misdirected energy the writer has as yet, or is ever likely to, come across. The most meticulous and unpractical German professor would admit that here he had met his match if he had to trace the numerous cross-lines which purport to show the patient's wanderings from his home H to P, a central bureau, to sanatorium M, to segregation institution N (inside or outside a hospital), to A, the general practitioner, to C, a hospital with a T.D., or to B, a T.D. outside a hospital affiliated to C, or to D, a hospital without a T.D., and so on over a large sheet.

Dr. King's idiosyncrasies in regard to charts must not be allowed to obliterate the good points of this book, but it seems a pity he should have thus obscured a reasonable scheme, and one which has the very important advantage over that passed by the L.C.C., inasmuch as it provides for the segregation of the advanced and infectious case. The text of some fifty pages describes fully the class of persons who will present themselves at various exchanges which are sub-divided very thoroughly. A good deal of repetition is due to the fact that insured persons are considered separately from uninsured persons, both in great detail. There is also included a scheme for dealing with tuberculosis among school children, with some facts in support. It is interesting to note that the author admits that there is evidence to prove that a "fair

* *Scheme for Dealing with Tuberculous Persons.* By D. Barty King, M.D. J. Bale, Sons, and Danielsson. 5s. net.

percentage of children are at present being diagnosed and notified as suffering from pulmonary tuberculosis on insufficient grounds." We believe that this is the case even if the rather wider term of thoracic tuberculosis be substituted for the above. No doubt the converse is also true, but to a far less extent.

Among certain other matters dealt with in this work are the duties of the tuberculosis officer and nurse, the medical officer of health, the general practitioner, and that newly discovered official, the "tuberculosis consulting physician," who presumably keeps his eye on the newly fledged T.O. An able criticism of the London County Council scheme is one of the best sections of this book. We hope that the labours of Dr. Barty King will be remembered when the necessary improvement of detail has to be faced.

